# Standardized trimodal histopathological examination for microplastic detection and tissue-level assessment in green mussels (*Perna viridis*) cultivated near an industrial estate in Rayong, Thailand

**DOI:** 10.14202/vetworld.2025.4212-4235

**Published:** 2025-12-31

**Authors:** Poramee Khongmeunrak, Patarakit Chongphaibulpatana, Thitichai Jarudecha, Wanat Sricharern, Khomson Satchasataporn, Pasavit Tapen, Suchanit Ngamkala

**Affiliations:** Department of Veterinary Nursing, Faculty of Veterinary Technology, Kasetsart University, 10900, Bangkok, Thailand

**Keywords:** environmental contamination, Green mussel, microplastics, Nile Red fluorescence, polarized light microscopy, Rayong coastline, trimodal histopathology

## Abstract

**Background and Aim:**

Microplastics (MPs) are persistent pollutants that build up in filter-feeding marine animals. Green mussels (*Perna viridis*), commonly eaten in Thailand, are effective bioindicators of water pollution. However, there are still limited standardized histopathological methods for detecting MPs and assessing lesions. This study aimed to develop a three-part histopathological exam using light microscopy, polarized light microscopy, and Nile Red (NR) fluorescence, and to analyze MP distribution and linked tissue effects in mussels collected from industrial shoreline areas in Rayong Province.

**Materials and Methods:**

Sixty-one mussels were collected from three coastal sites near an industrial estate. Target tissues were processed using a novel isopropanol-based, xylene-free protocol to prevent MP loss. Serial sections were stained with hematoxylin–eosin, evaluated under polarized light, and analyzed for NR fluorescence. Particle confirmation required co-localization across all three methods. Histopathological lesions were scored semi-quantitatively, and statistical associations were assessed using Fisher’s exact test, McNemar’s test, and generalized estimating equations.

**Results:**

MP prevalence was 59.0% using light and polarized microscopy, 44.3% with NR fluorescence, and 39.3% with trimodal confirmation. The digestive tract showed the highest MP accumulation (64.1%), followed by the gills (33.3%) and the digestive glands (15.4%). Most MPs were irregular fragments (91.67%), mainly 10–100 μm in size. Mussel size was not significantly linked to MP contamination (p = 0.224). Notably, 88.9% of MP-positive tissues showed no observable histopathological changes; only 11.1% had mild to moderate lesions, including epithelial cell damage and hemocyte infiltration in digestive tracts, gills, and digestive glands.

**Conclusion:**

The standardized trimodal histopathological approach offers a reliable, fast, and xylene-free method for MP detection in *P. viridis*. Digestive tracts, gills, and digestive glands are the most informative tissues for biomonitoring. Although many tissues did not show obvious lesions, the presence of mild pathological changes highlights the potential for sublethal effects in chronically exposed populations. This method improves diagnostic accuracy by reducing false positives and provides a consistent framework for MP surveillance in industrial coastal zones.

## INTRODUCTION

Plastics are extensively used in daily life across many sectors, including packaging, electronics, automotive manufacturing, and the textile industry [1, 2]. Over time, discarded plastics break down into microplastics (MPs), which can originate as primary MPs, such as industrial pellets or microbeads in personal care products [3, 4], or as secondary MPs, formed through environmental degradation caused by ultraviolet radiation, heat, mechanical abrasion, and chemical or biological processes [5, 6]. Ineffective waste management further speeds up MP buildup in soil, rivers, and marine ecosystems [7–9]. Aquatic organisms, located centrally in the food web, easily ingest MPs, aiding their transfer to higher trophic levels, including humans [10, 11]. Ingested MPs may block intestinal passages and stay in various tissues, while experimental studies have shown that MP exposure causes oxidative stress, immune dysregulation, cellular and DNA damage, and changes in tissue structure in marine species and humans [10, 12, 13]. According to the World Health Organization, daily human MP intake is estimated to range from 0.6 micrograms to 700 milligrams [4], highlighting growing global concern about MP contamination in the environment and in seafood.

In Thailand, multiple studies have confirmed MP contamination in various aquatic species [14, 15], with especially high detection rates in green mussels (*Perna viridis*) [16]. Mussels are both economically and nutritionally important across the country [17] and serve as effective bioindicators of MP pollution because of their filter-feeding behavior, limited mobility, and tolerance to high pollutant levels [18]. Mussel farming sites along the Rayong coast are located near major industrial estates, which have been identified as consistent sources of MP pollution [19–21]. Polyethylene (PE) is the main type of MP found in beach sand and coastal seawater in this area [20]. Recent findings in 2024 showing MP contamination in market-sourced green mussels from Rayong [10] have heightened concerns about industrial discharges and the potential risks to consumers.

Various analytical approaches have been employed to investigate MP contamination in shellfish [10, 18]. Chemical digestion is still widely used for isolating MPs from mussel, clam, cockle, and oyster tissues in Thailand [10, 16, 17]. Visual identification methods, such as stereomicroscopy, scanning electron microscopy (SEM), and Nile Red (NR) staining combined with micro–Fourier transform infrared spectroscopy (μ-FTIR), are used to assess morphology and identify polymers. However, studies on the histopathological localization of MPs and related tissue lesions in mussels from industrial zones remain limited [16]. Recently, an integrated trimodal histopathological approach, combining light microscopy [22], polarized light microscopy [13], and NR–based fluorescence microscopy [23, 24], has been proposed to improve MP detection and physical characterization in mussel tissues [25]. This approach provides a standardized framework for examining MP distribution and tissue changes in green mussels from Rayong Province.

Despite growing concerns about MP pollution in Thailand’s coastal areas, significant gaps remain in understanding the tissue-level distribution, localization, and histopathological impacts of MPs in *P. viridis*, especially in regions affected by industry and local communities. Previous research in Thailand has mostly used chemical digestion and spectroscopic analysis to measure MPs in mussels and other bivalves, mainly focusing on prevalence and polymer types. However, these methods offer limited insights into the microscopic interactions between MPs and host tissues, their *in situ* shapes, and whether MPs cause subtle or tissue changes. Additionally, available histopathological studies are few, lack standardized methods, and often do not confirm MP identity using multiple imaging techniques, which increases the chance of misclassifying non-polymeric particles. Importantly, no research in Rayong Province, a known hotspot for industrial MP contamination, has employed a validated, multimodal histopathological method to analyze MPs within tissue microenvironments. Therefore, there is an urgent need for a standardized, dependable, xylene-free histopathological technique that can detect MPs, reduce particle loss during processing, distinguish true MPs from biological or inorganic artifacts, and allow the assessment of tissue lesions and co-localization patterns.

To address these gaps, the present study aimed to develop and standardize a trimodal histopathological examination, integrating light microscopy, polarized light microscopy, and NR fluorescence microscopy, for the rapid and accurate detection of MPs within *P. viridis* tissues collected from mussel farms near industrial estates in Rayong Province, Thailand. This study also sought to characterize the distribution, size, and morphology of MPs across key tissues (digestive tracts, gills, digestive glands, gonads, and adductor muscles) and to evaluate potential MP-associated histopathological changes using a semi-quantitative scoring system. By validating an isopropanol-based, xylene-free processing workflow and using low-density polyethylene (LDPE)-spiked positive controls, the study aimed to establish a reproducible and contamination-controlled diagnostic framework that enhances MP detection accuracy and provides foundational data for future biomonitoring, ecological risk assessment, and seafood safety evaluations.

## MATERIALS AND METHODS

### Ethical approval

The experimental protocol for this study was reviewed and approved by the Institutional Animal Care and Use Committee of Kasetsart University, Bangkok, Thailand (Approval ID: ACKU66-VTN-013). All procedures involving green mussels (*P. viridis*), including field collection, transport, handling, euthanasia, tissue sampling, and histopathological processing, were carried out in accordance with the institutional guidelines for the care and use of animals in research and the biosafety regulations of Kasetsart University and the National Research Council of Thailand. Mussels were obtained from local farms and seafood markets with owners’ permission and were not sourced from protected or endangered populations. Euthanasia was performed by immersion in 10% ethanol for at least 10 min, following the American Veterinary Medical Association (AVMA) Guidelines for the Euthanasia of Animals (2020), to minimize pain and distress before tissue collection.

### Study period and location

The present study was conducted from August 2023 to June 2025 at the histopathology laboratory, Faculty of Veterinary Technology; the Department of Anatomy, Faculty of Veterinary Medicine; and the Department of Zoology, Faculty of Science, Kasetsart University, Bangkok, Thailand.

### Sample collection and processing

The sample size was determined using a formula for an infinite population proportion, with 80% statistical power, an alpha level of 0.05, an expected prevalence (p) of 0.84, and a margin of error (d) of 0.16, based on previous studies [16, 26].

**Figure 1:**
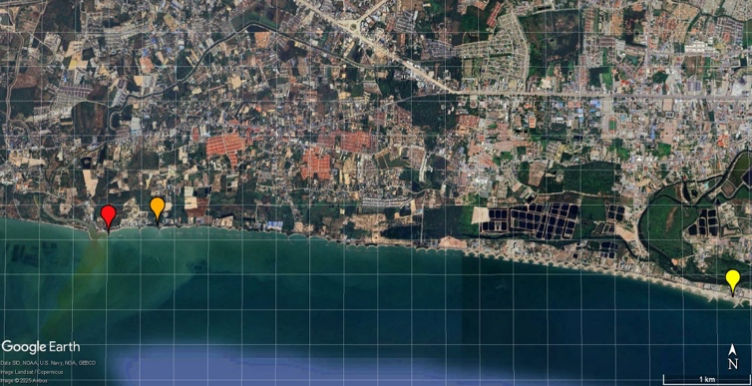
The site for fresh green mussel sample collection; Takuan beach (red pin), Suchada beach (orange pin), and Seang Chan beach (yellow pin) located near the Industrial Estate of Rayong Province. Scale bar = 1 km. Source: Adapted from Google Earth, Imagery Date: 3/31/2024.

Between August–September 2023 and March–April 2024, a total of 61 fresh green mussels (*P. viridis*) were randomly collected from local farms and fish markets at three coastal sites along Rayong’s industrial shoreline (Figure 1):

- Takuan Beach: 12°40’8.86”N, 101°10’54.18”
- Suchada Beach: 12°40’11.88”N, 101°11’13.04”
- Seang Chan Beach: 12°39’45.00”N, 101°14’53.09”

Simultaneously, seawater temperature, salinity, and pH were recorded at each site:

- Takuan Beach: 31.63 ± 0.06°C, 28.44 ± 0.04 ppt, pH 8.22 ± 0.02
- Suchada Beach: 30.03 ± 0.115°C, 28.61 ± 0.35 ppt, pH 8.30 ± 0.015
- Seang Chan Beach: 30.13 ± 0.06°C, 28.47 ± 0.05 ppt, pH 8.00 ± 0.04

Collected mussels were wrapped in aluminum foil, transported on ice to the local animal hospital, rinsed with deionized (DI) water, and euthanized by immersion in 10% ethanol for at least 10 minutes following AVMA guidelines [27]. Gross lesions and physical measurements (shell dimensions, visceral mass size, and wet weight) were recorded. Tissues were dissected and fixed in Davidson’s fixative [28] at 25°C for 24–72 h, then transferred to the histopathology laboratory at Kasetsart University.

Twenty mussels were designated as positive controls. After euthanasia and overnight fixation, each mussel was injected with 2–4 mL of a suspension containing 500 μm low-density polyethylene (LDPE) MP powder (250 mg/100 mL Davidson’s fixative) [13].

### Histopathological analysis

Fixed tissues (digestive tracts, digestive glands, gills, adductor muscles, and gonads) and positive controls were washed under running water for 1 h and trimmed to 1 × 1 cm. Tissue processing was performed using an automated vacuum processor (Shandon Citadel 2000, Thermo Fisher Scientific, Waltham, MA, USA), replacing xylene with isopropyl alcohol (IPA) (Avantor, Inc., Macron Fine Chemicals™, Radnor, Pennsylvania, USA) throughout dehydration and clearing to prevent MP loss [28]. The modified protocol followed sequential IPA concentrations.

- 70% IPA: 1 × 1 h
- 80% IPA: 1 × 1 h
- 90% IPA: 2 × 1 h
- 100% IPA: 3 × 1 h

Tissues were then vacuum-infiltrated with paraffin (Histoplast PE, Epredia, Portsmouth, NH, USA) at 56°C–57°C overnight and embedded using the Shandon Histocentre 3 (Thermo Fisher Scientific). Paraffin blocks were serially sectioned at 5 μm (3 serial sections per plane, 6 planes per sample) using a rotary microtome (RM2245, Leica Biosystems, Deer Park, IL, USA). Ribbons were floated at 40°C–42°C. Xylene-free deparaffinization and rehydration were performed using heating and immersion in graded IPA, respectively. Primary sections were HE-stained and mounted with 50% glycerol for MP detection. Secondary sections were stained with NR working solution (1 μg/mL in methanol) for 10 min [23], rinsed, and mounted using 50% glycerol (RI = 1.395).

### MP detection and characterization

Trimodal examination was performed to confirm the same particle across serial sections:

- Light microscopy with polarized filter (CX33, Olympus; 10×; exposure 38 ms; gain 0.13) for HE-stained tissues and birefringence.
- NR fluorescence microscopy (Axiolab A1, Carl Zeiss Microscopy GmbH, Jena, Germany; fluorescein Isothiocyanate (FITC): 495/517 nm, Rhodamine (Rhod): 551/573 nm)

LDPE-spiked control tissues were used to validate detection across modalities.

Particles were identified as MP candidates if they showed morphological differences from native tissue, remained unstained in HE, were birefringent under polarized light, and emitted green or orange fluorescence under FITC and Rhod filters. MPs were classified by:

- Size: ≤1 μm, 1–10 μm, 10–100 μm, >100 μm [29]
- Shape: fibers (thin, elongated) and fragments (irregular) [16]

Semi-quantitative MP counts were recorded as particles per section.

### Histopathological evaluation

HE-stained slides were independently evaluated by three blinded pathologists at 40× magnification. Lesions were graded using a four-level semi-quantitative scale (Table 1): 0 = no observable histopathological lesion, 1 = mild, 2 = moderate, 3 = severe [30–35]. MPs within body cavities without tissue contact were scored as 0.

**Table 1:** The descriptive information of pathological change and corresponding scoring criteria [32, 34, 35].

Tissue types	Histopathological lesion score	Description	Photo of the negative control (40× magnification, scale bar 20 μm)
Digestive tracts	No observable histopathological changes (0)	No hemocyte infiltration Great alignment of the epithelial cells Normal tubular lumen size No evidence of tissue destruction was observed	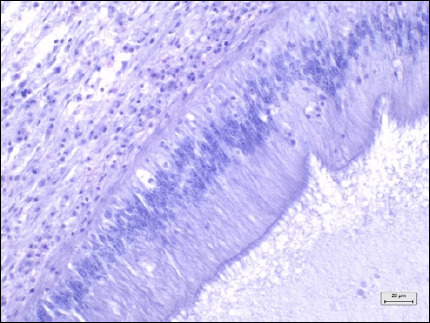
	Mild (1)	Hemocyte infiltration for 1/3 of section Mild decrease in the number of epithelial cells and glandular cells Mild destruction of the tubular structure No evidence of tissue destruction was observed	
	Moderate (2)	Hemocyte infiltration for 2/3 of the sections Moderate decrease in the number of epithelial cells and glandular cells Moderate destruction of the tubular structure Evidence of tissue destruction	
	Severe (3)	Hemocyte infiltration of more than 2/3 of the section Severe decrease in the number of epithelial cells and glandular cells Severe destruction of the tubular structure Evidence of tissue destruction	
Digestive glands	No observable histopathological changes (0)	No hemocyte infiltration Normal size, thickness, and structure of digestive (clear-oval) and basophilic (pyramid) cells Clear digestive lumens No evidence of tissue destruction was observed	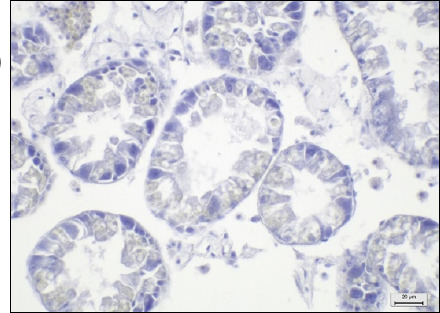
	Mild (1)	Hemocyte infiltration for 1/3 of section Abnormal size, thickness, and structure of digestive (clear-oval) and basophilic (pyramid) cells Digestive lumen atrophy or hyperplasia for 1/3 of section No evidence of tissue destruction was observed	
	Moderate (2)	Hemocyte infiltration for 2/3 of the sections Abnormal size, thickness, and structure of digestive (clear-oval) and basophilic (pyramid) cells Digestive lumen atrophy or hyperplasia in 2/3 of the sections Evidence of tissue destruction	
	Severe (3)	Hemocyte infiltration of more than 2/3 of the section Abnormal size, thickness, and structure of digestive (clear-oval) and basophilic (pyramid) cells Digestive lumen atrophy or hyperplasia for the whole section Evidence of tissue destruction Evidence of fibrosis or granuloma	
Gills	No observable histopathological changes (0)	No hemocyte infiltration Clear gill filament structure Number and thickness of epithelial cells Normal thickness of the hemolymph sinus	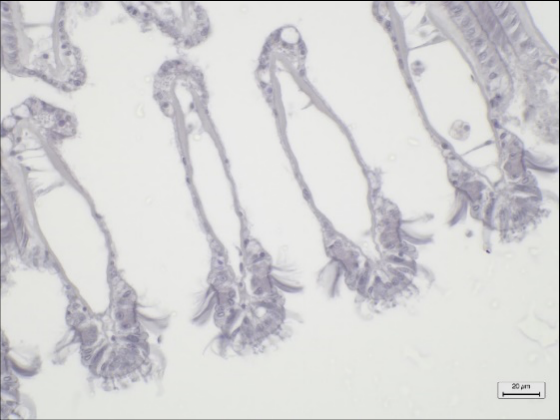
	Mild (1)	Hemocyte infiltration for 1/3 of section Mild decrease in the number and thickness of epithelial cells Mild thickness of the hemolymph sinus	
	Moderate (2)	Hemocyte infiltration for 2/3 of the sections Moderate decrease in the number and thickness of epithelial cells Moderate hemolymph sinus thickness Evidence of gill filament fusion	
	Severe (3)	Hemocyte infiltration of more than 2/3 of the section Complete loss of gill filaments Severe hemolymph sinus thickness	
Gonads (male)	No observable histopathological changes (0)	High spermatocyte density around the gonad gland Spermatogenesis around the gonad gland	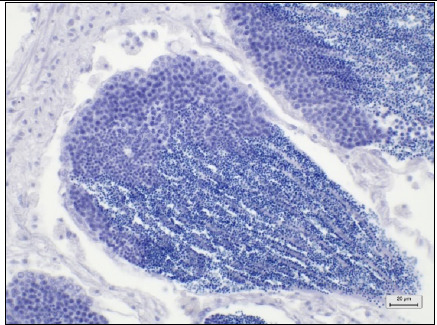
	Mild (1)	Lost density of spermatocytes for 1/3 of the gonad gland section Evidence of spermatogenesis for 2/3 of the gonad	
	Moderate (2)	Lost density of spermatocytes for 2/3 of the gonad gland Evidence of spermatogenesis for 1/3 section of gonad	
	Severe (3)	Complete loss of spermatocyte density of gonad gland No evidence of spermatogenesis	
Gonads (Female)	No observable histopathological changes (0)	High-density of oocytes around the GC Oogenesis around the gonad gland	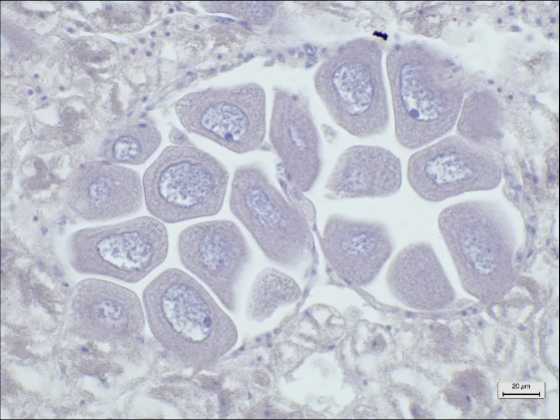
	Mild (1)	Lost density of oocytes for 1/3 of the gonad gland section Evidence of oogenesis for 2/3 of the gonad	
	Moderate (2)	Lost density of oocytes for 2/3 section of gonad gland Evidence of oogenesis for 1/3 section of gonad	
	Severe (3)	Complete loss of oocyte density of gonad gland No evidence of oogenesis	
Muscles	No observable histopathological changes (0)	Normal myocyte structure No hemocyte infiltration No ceroid bodies	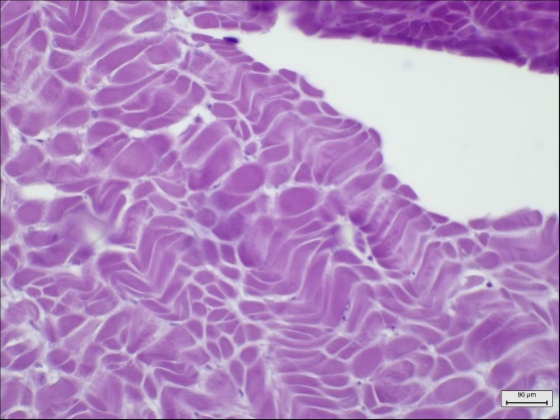
	Mild (1)	Evidence of myocyte degeneration No hemocyte infiltration No ceroid bodies	
	Moderate (2)	Evidence of myocyte degeneration Hemocytes infiltration No ceroid bodies	
	Severe (3)	Evidence of myocyte degeneration Evidence of hemocyte infiltration Evidence of the ceroid bodies	
Body cavities	No observable histopathological changes (0)	MP evidence in body cavities without tissue contact	

### Statistical analysis

Data were analyzed using STATA 17.0 (StataCorp, College Station, TX, USA) with 80% power and a 95% confidence interval. Continuous variables were assessed using histograms and the Shapiro–Wilk test; non-continuous variables were evaluated based on the central limit theorem.

Comparisons among the three detection methods were conducted using the McNemar test [36]. Associations between MP contamination and mussel size were assessed with Fisher’s exact test [37] and pairwise post-hoc tests. Significance was set at p < 0.05.

Generalized Estimating Equations (GEEs) [38] were used to calculate adjusted odds ratios for:

- Mussel size vs. MP contamination
- Tissue type vs. MP distribution

A logistic link and binary family with exchangeable correlation were used. Model fit was evaluated using Quasi-likelihood Information Criterion values. Inter-observer reliability was assessed using intraclass correlation coefficient (ICC), with values >0.7 indicating good agreement [39].

### Quality assurance and quality control

All procedures adhered to strict MP contamination control measures. Only nonplastic equipment (glass, metal, aluminum) was used. All materials were pre-rinsed, covered with aluminum foil, and handled by personnel wearing nitrile gloves, cotton lab coats, and masks. DI water was pre-filtered before use.

Samples were wrapped in aluminum foil, transported on ice, rinsed, and processed in a fume hood to reduce airborne contamination. Blank slides were used as negative controls. A particle was confirmed as MP only when identified at the same anatomical location across all three trimodal techniques and matched the LDPE control characteristics.

## RESULTS

### MP detection

**Figure 2:**
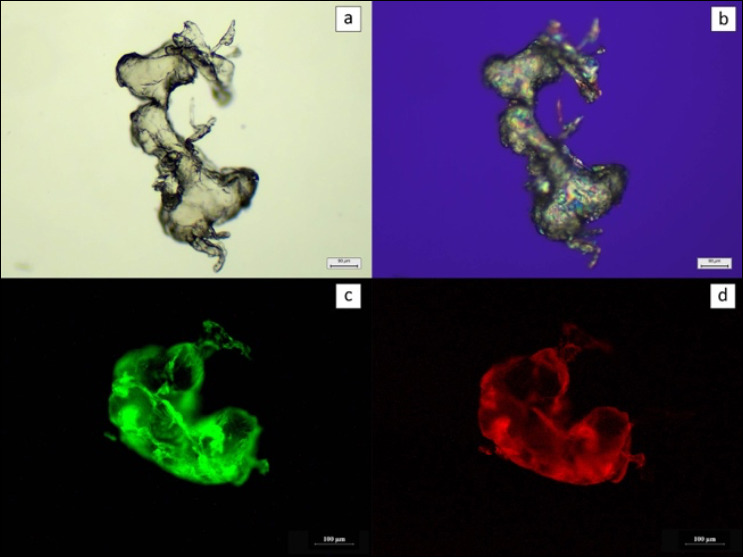
LDPE particle detection (a) under a light microscope at 10× magnification; (b) under a polarized light microscope at 10x magnification; (c) NR-stained under a fluorescence microscope using fluorescein isothiocyanate and (d) Rhod filters at 10x magnification. Scale bar: (a andb) 90 μm and (c and d) 100 μm.

**Figure 3:**
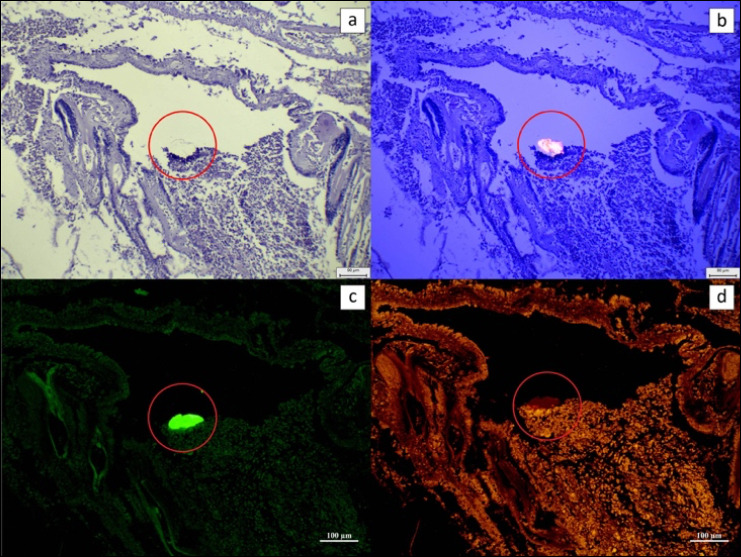
The LDPE particle detection in mussel mantle at 10× magnification. (a) HE-stained tissue under a light microscope, (b) HE-stained tissue under a polarized light microscope, (c) NR-stained tissue under a fluorescence microscope using FITC, and (d) Rhod filters. The red circle shows the LDPE particles in the tissue. Scale bar (a and b) 90 μm and (c and d) 100 μm.

LDPE particles used as external controls were initially examined using the trimodal detection approach (Figure 2). Under light microscopy, LDPE particles appeared unstained, while polarized light microscopy showed distinct birefringence. After NR staining, fluorescence microscopy confirmed LDPE identity through green fluorescence under the FITC filter and orange fluorescence under the Rhod filter. Positive control tissues injected with LDPE displayed identical optical characteristics across all three detection methods (Figure 3).

Subsequently, trimodal histopathological examination was performed on 61 *P. viridis* tissue samples. The detection rates with the three techniques were as follows:

- Light microscopy (HE-stained): 59.0% (36/61; 95% CI: 45.7–71.4)
- Polarized light microscopy: 59.0% (36/61; 95% CI: 45.7–71.4)
- NR fluorescence microscopy: 44.3% (27/61; 95% CI: 31.5–57.5)
- Trimodal confirmation: 39.3% (24/61; 95% CI: 27.1–52.7)

**Figure 4:**
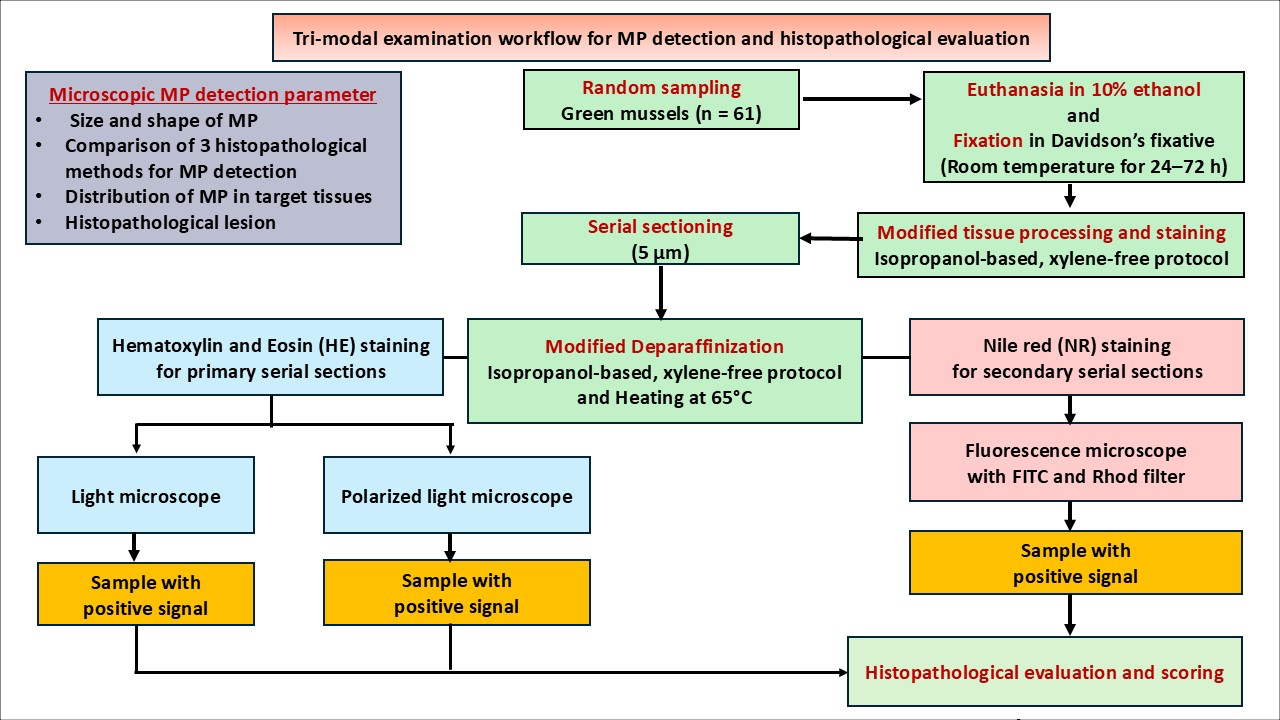
The schematic of trimodal examination for MP detection workflow.

Light and polarized microscopy detected MPs in the same 36 samples. However, 24 of the NR-positive samples overlapped with trimodal-confirmed identifications. The entire trimodal detection workflow is shown in Figure 4.

MP abundance was evaluated using two metrics: total particle count and median particles per section (Interquartile Range; IQR).

- Light and polarized microscopy: 97 total particles; 2(2) particles/section
- Fluorescence microscopy: 53 particles; 2(1) particles/section
- Trimodal detection: 48 particles; 2(1) particles/section

Detailed counts by tissue type and detection technique are shown in Table 2.

**Table 2:** Statistical summarization of MP counts by tissue type and microscopy method.

Tissue	MP counts per section, n = Median (IQR)									

	Light microscopy (HE) "A" n = 36		Polarized light microscopy (HE) "B" n = 36		Fluorescence microscopy (NR) "C" n = 27		Trimodal examination “A, B, or C” n = 24		Any histopathological techniques “A, B, or C” n = 39	

	Number of MP per tissue (particle)	Median (IQR) of MP per section	Number of MP per tissue (particle)	Median (IQR) of MP per section	Number of MP per tissue (particle)	Median (IQR) of MP per section	Number Of MP per tissue (particle)	Median (IQR) of MP per section	Number of MP per tissue (particle)	Median (IQR) of MP per section
Digestive tracts	55	1(2)	55	1(2)	27	1(1)	24	1(1)	58	1(1.5)
Digestive glands	7	1(1)	7	1(1)	6	1.5(1)	5	2(1)	8	1(1)
Gills	19	1(1)	19	1(1)	10	1(1)	9	1(1)	20	1(1)
Male gonads	3	1	3	1	1	1(N/A)	1	1(N/A)	3	1
Female gonads	3	1.5(1)	3	1.5(1)	2	2(N/A)	2	2(N/A)	3	1.5(1)
Adductor muscles	2	1	2	1	1	1(N/A)	1	1(N/A)	2	1
Body cavities	8	2(1)	8	2(1)	6	2(2)	6	2(2)	8	2(1)
All samples	97	2(2)	97	2(2)	53	2(1)	48	2(1)	102	2(2)

MP = Microplastic, IQR = Interquartile range, n = positive sample of MP-contaminated tissues, A = light microscope (HE), B = polarized light microscope (HE), C = fluorescence microscope, NR = Nile Red, A, B, and C = trimodal examination, and A, B, or C = any histopathological technique. IQR = 75^th^ percentile – 25^th^ percentile. N/A = Not applicable.

### Characterization of MPs

A total of 48 MP particles were confirmed through trimodal examination. Size classification (median, IQR) indicated:

- >100 μm: 3 particles; 293.94(225.16) μ
- 10–100 μm: 44 particles; 40.70(26.39) μ
- 1–10 μm: 1 particle; 9.38 μ
- ≤1 μm: none detected

Shape analysis showed that most MPs were irregular fragments (44 particles; 91.7%), while fibers made up 8.3% (4 particles).

### Distribution of MPs

The 61 mussels were categorized into three size groups:

- Small: 50.00–59.99 mm
- Medium: 60.00–69.99 mm
- Large: 70.00–79.99 mm

Overall, MPs were found in 63.9% (39/61) of mussels. Medium-sized mussels had the highest proportion of MP-positive samples (51.3%; 20/39), followed by small (28.2%; 11/39) and large mussels (20.5%; 8/39).

MPs were distributed across various tissues:

- Digestive tracts: 64.1% (25/39)
- Gills: 33.3% (13/39)
- Digestive glands: 15.4% (6/39)
- Body cavities: 10.3% (4/39)
- Male gonads: 7.7% (3/39)
- Female gonads: 5.1% (2/39)
- Adductor muscles: 5.1% (2/39)

A summary of MP counts per tissue is provided in Table 2.

### Histopathological lesion analysis

**Figure 5:**
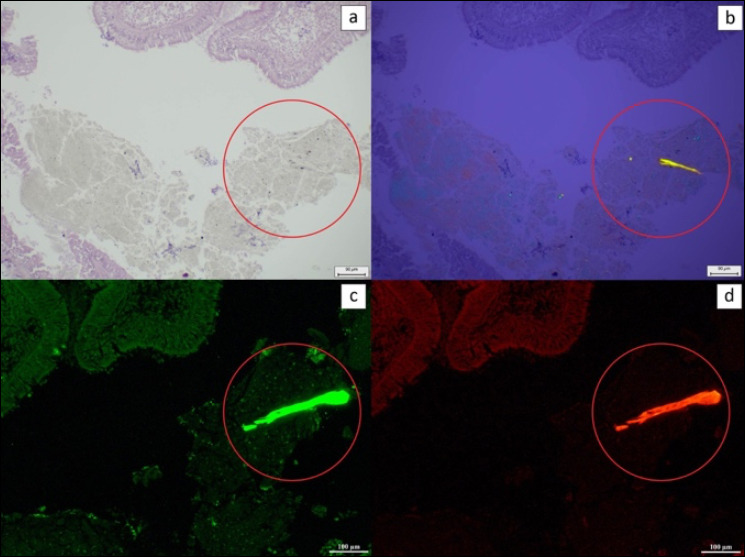
The MP fibers contaminated in mussel digestive tracts with no observable histopathological alteration with MP co-localization nearby tissues at 10× magnification. (a) HE-stained tissue under a light microscope, (b) HE-stained tissue under a polarized light microscope, (c) NR-stained tissue under a fluorescence microscope, using FITC, and (d) Rhod filters. The red circle shows the MP fibers in the tissue. Scale bar (a and b) 90 μm and (c and d) 100 μm.

**Figure 6:**
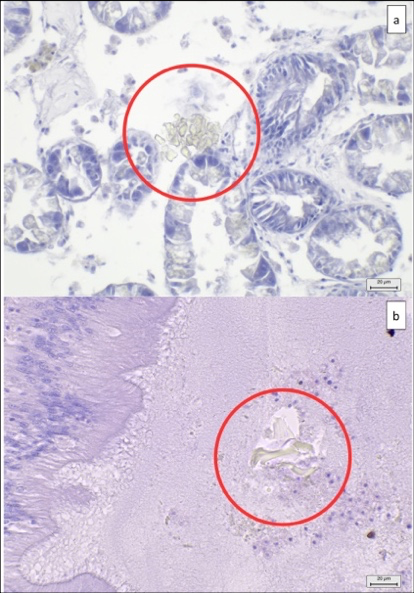
Histopathology of the digestive tracts and glands with no observable histopathological alteration with MP co-localization near tissues (HE) at 40× magnification. (a) Digestive glands showed normal digestive cell structure, with no hemocyte infiltration adjacent to the MP particle (red circle) and (b) MP particle identified in the digestive lumens, with normal digestive lumen epithelial structure without hemocyte infiltration. Scale bar = 20 μm.

Gross examination showed no macroscopic lesions in the shells or internal soft tissues. Among the 39 MP-positive mussel samples examined microscopically, 88.9% (32/36) showed no observable histopathological changes, despite clear co-localization of MPs in tissues such as the digestive tracts (Figure 5) and digestive glands (Figure 6).

**Figure 7:**
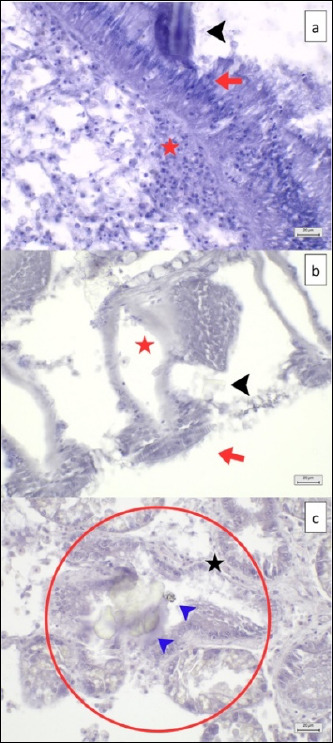
MP co-localization in digestive tracts, gill filaments, and digestive glands with mild histopathological lesions (HE) at 40× magnification. (a) Fiber-shaped MP particle (black arrowhead) in digestive lumens, resulting in epithelial cell damage (red arrow) and hemocyte infiltration (red star) within the tissues adjacent to epithelial cells, (b) MP particle (black arrowhead) attached to gill filaments, resulting in cilia destruction (red arrow) and hemocyte infiltration (red star), and (c) MP particle (blue arrowhead) in digestive glands, resulting in digestive lumen atrophy (red circle) with loss of digestive cells (black star). Scale bar = 20 μm.

Only 11.1% (4/36) of MP-positive samples displayed microscopic lesions:

- Digestive tracts: 25% (1/4) – mild lesions
- Digestive glands: 25% (1/4) – mild; and one sample showing moderate lesions
- Gills: 25% (1/4) – mild lesions (Figure 7)

**Figure 8:**
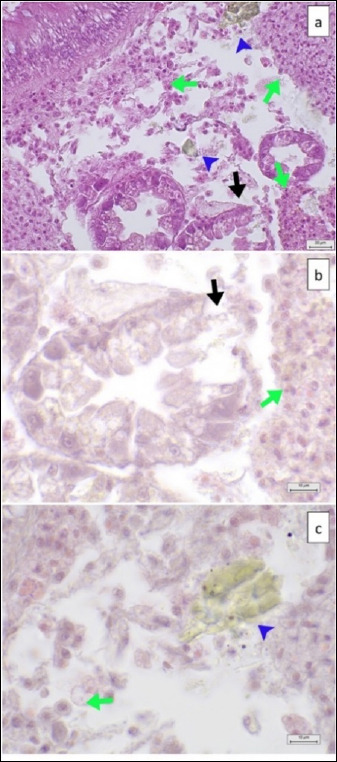
MP particles co-localization in digestive glands (HE) with moderate histopathological lesions. (a) MP particles (blue arrowhead) in digestive glands (at 40× magnification), resulting in lumen destruction (black arrow), tissue destruction with hemocyte infiltration (green arrow), (b) lumen destruction (black arrow) and tissue destruction with hemocyte infiltration (green arrow) (at 100× magnification), and (c) MP particles (blue arrowhead) co-localization near tissue, showing tissue destruction with hemocyte infiltration (green arrow) (at 100× magnification). Scalebar = (a) 20 μm and (b) 10 μm.

Moderate lesions (25%; 1/4) were found exclusively in the digestive glands (Figure 8). Inter-observer reliability for lesion scoring was high (ICC >0.7).

### Statistical analysis

Using the McNemar test (Table 3), HE-stained light microscopy and polarized light microscopy exhibited the highest MP detection rates (59.0%; 95% CI: 45.7–71.4). NR fluorescence microscopy detected significantly fewer MP-positive samples (44.3%; p = 0.035), and the trimodal approach identified the lowest proportion (39.3%; p = 0.001).

**Table 3:** McNemar test results of the comparison of appropriate MP detection methods.

Histopathological techniques	Percentage of suspected MPs (Positive sample/Total sample)	95% CI Positive percentage
Light microscopy (HE)	59.0(36/61)^A^	45.7–71.4
Polarized light microscopy (HE)	59.0 (36/61)^B^	45.7–71.4
Fluorescence microscopy (NR)	44.3 (27/61)^C^	31.5–57.5
Trimodal examination	39.3 (24/61) ^D^	27.1–52.7

MP = Microplastic. Statistical analysis was performed using the McNemar test with a p-value. A vs B showed a p-value of 1.000, A vs C and B vs C showed a p-value of 0.035, C vs D showed a p-value of 0.250, A vs D and B vs D showed a p < 0.001. p-values less than 0.05 were considered significant. CI = Confidence interval, HE = Hematoxylin and Eosin-stained tissues. NR = Nile red-stained tissues.

Comparisons revealed:

- Light vs. polarized microscopy: no difference (p = 1.000)
- Light vs. NR fluorescence: significant difference (p = 0.035)
- Polarized vs. NR fluorescence: significant difference (p = 0.035)
- Light vs. trimodal: significant difference (p = 0.001)
- Polarized vs. trimodal: significant difference (p = 0.001)
- Trimodal vs. NR fluorescence: no difference (p = 0.250)

The Fisher’s exact results (Table 4) revealed MP detection did not differ significantly among mussel size groups (p = 0.224).

**Table 4:** Fisher’s exact results of the relationship between MP contamination and green mussel size.

Size	MP contamination percentage (%) (Positive sample/Total sample in each group)	95% CI of the MP contamination	p-value
Small	84.6% (11/13)	54.5–98.1	0.224
Medium	57.1% (20/35)	39.3–73.7	
Large	61.5% (8/13)	31.6–86.1	

MP = Microplastic, CI = Confidence interval, Statistical analysis by Fisher’s exact analysis, p < 0.05 was considered significant.

GEE analysis (Table 5) showed:

**Table 5:** Risks (odds ratio) of the MP contamination between green mussel size (n = 61) under a generalized estimating equations analysis.

Group	Odds ratio	95% Confidence interval	p-value
Adjusted by different types of mussel tissues			
Small size (n = 13)	1	Reference	
Medium size (n = 35)	0.48	0.23–0.99	0.047
Large size (n = 13)	0.53	0.23–1.22	0.135
Medium size (n = 35)	1	Reference	
Small size (n = 13)	2.07	1.01–4.26	0.047
Large size (n = 13)	1.09	0.51–2.37	0.816

MP = Microplastic, Statistical analysis by generalized estimating equation analysis, p < 0.05 was considered a significant difference. n = number of mussels in each group.

- Small mussels had significantly higher odds of MP contamination compared to medium mussels (OR = 0.48; 95% CI: 0.23–0.99; p = 0.047).
- Comparisons between other size groups showed no significant differences.

Tissue-based GEE analysis (Table 6) showed significant differences in MP distribution when the digestive tract served as the reference organ. Using gills as the reference revealed significant differences in all tissues except the digestive glands (p = 0.092).

**Table 6:** Risks of MP contamination among green mussel tissues (n = 61) under a generalized estimating equation analysis.

Tissue types	Number of samples contaminated with microplastic	OR	95% Confidence interval	p-value
**Digestive tracts**	25	1	Reference	
Digestive glands	6	0.15	0.05 - 0.44	0.001
Gills	13	0.38	0.17–0.85	0.019
Gonads (M)	3	0.07	0.02 - 0.24	0.000
Gonads (F)	2	0.05	0.01 - 0.21	0.000
Adductor muscles	2	0.05	0.01 - 0.21	0.000
Body cavities	4	0.10	0.03 - 0.30	0.000
**Digestive glands**	6	1	Reference	
Digestive tracts	25	6.57	2.27 - 19.00	0.001
Gills	13	2.51	0.85–7.35	0.092
Gonads (M)	3	0.47	0.15–1.43	0.186
Gonads (F)	2	0.31	0.05–1.70	0.177
Adductor muscles	2	0.31	0.05–1.71	0.178
Body cavities	4	0.64	0.18–2.23	0.485
**Gills**	13	1	Reference	
Digestive tracts	25	2.61	1.17 - 5.83	0.019
Digestive glands	6	0.40	0.13 - 1.16	0.092
Gonads (M)	3	0.19	0.05 - 0.67	0.010
Gonads (F)	2	0.12	0.02–0.61	0.010
Adductor muscles	2	0.12	0.02–0.62	0.011
Body cavities	4	0.25	0.07–0.91	0.035
**Gonads (M)**	3	1	Reference	
Digestive tracts	25	13.93	4.19–46.33	0.000
Digestive glands	6	2.12	0.70–6.46	0.186
Gills	13	5.33	1.48–19.16	0.010
Gonads (F)	2	0.65	0.10–4.32	0.659
Adductor muscles	2	0.65	0.10–4.32	0.659
Body cavities	4	1.36	0.35–5.29	0.658
**Gonads (F)**	2	1	Reference	
Digestive tracts	25	21.30	4.78–94.83	0.000
Digestive glands	6	3.24	0.59–17.92	0.177
Gills	13	8.15	1.64–40.56	0.010
Gonads (M)	3	1.53	0.23 - 10.09	0.659
Adductor muscles	2	1.00	0.13–7.77	1.000
Body cavities	4	2.08	0.34 - 12.56	0.425
**Adductor muscles**	2	1	Reference	
Digestive tracts	25	21.30	4.68–96.83	0.000
Digestive glands	6	3.24	0.58–17.98	0.178
Gills	13	8.15	1.62–40.98	0.011
Gonads (M)	3	1.53	0.23 - 10.10	0.659
Gonads (F)	2	1.00	0.13–7.77	1.000
Body cavities	4	2.08	0.34 - 12.58	0.426
**Body cavities**	4	1	Reference	
Digestive tracts	25	10.25	3.32–31.63	0.000
Digestive glands	6	1.56	0.45–5.44	0.485
Gills	13	3.92	1.10 - 14.00	0.035
Gonads (M)	3	0.73	0.19–2.86	0.658
Gonads (F)	2	0.48	0.08–2.91	0.425
Adductor muscles	2	0.48	0.08–2.91	0.426

MP = Microplastic, Statistical analysis using generalized estimating equations; a p-value less than 0.05 was considered significant. n = number of microplastic-contaminated samples in each mussel tissue. OR = Odds ratio, CI = Confidence interval, M = Male, F = Female.

## DISCUSSION

### Histopathological techniques for the diagnosis of MP

#### Positive controls and validation of trimodal examination

Positive controls were prepared by infiltrating the green mussels with LDPE suspended in Davidson’s fixative (250 mg/100 mL). This process provided controlled in-tissue MP samples essential for generating reliable calibration and training datasets, which help validate and strengthen the reliability of the trimodal examination.

The tissue-processing and staining procedures in the present study were independently modified from the previously described protocol [28]. During tissue processing, xylene was replaced with IPA to enhance MP preservation, and the durations of the dehydration and paraffin-infiltration steps were extended to improve tissue conditioning. In the staining procedure, only the substitution of xylene with IPA was implemented.

Subsequently, positive control LDPE particles were identified through tripartite histopathological examination (light, polarized light, and NR-stained fluorescence microscopy), each revealing unique optical characteristics. LDPE appeared as unstained particles under light microscopy, birefringent under polarized light microscopy, and green and orange fluorescent particles under the FITC and Rhod filters, respectively, after NR staining. LDPE-spiked control tissues exhibited the same features as LDPE powder particles.

#### Light microscopy for MP screening

MP contamination was examined in various green mussel tissues using HE-stained samples viewed under a light microscope to evaluate MP distribution and possible histopathological changes. Light microscopy is a common technique used for initial examination and assessment of tissue alterations. The unstained suspected MP particles appeared in various sizes and shapes. Furthermore, the current study replaced xylene with IPA in tissue processing steps such as paraffin infiltration, deparaffinization, and used aqueous mounting media since xylene or strong organic solvents could dissolve MPs. Furthermore, the different staining methods used under these conditions produced effective results, similar to a previous study on preserved MP-contaminated mussel tissues [28]. Compared to the previous work, using IPA in tissue processing and staining showed no statistical difference in section quality, tissue structure, or staining when compared with xylene in lung tissues [40].

These findings show that IPA is suitable for histopathological studies of MPs within mussel tissues [28] and is also operator- and environmentally friendly [40]. In the current results, MP particles were identified using light microscopy. Light microscopy has previously been employed to detect MP particles, specifically polystyrene (PS), in various tissues such as digestive tracts, digestive glands, gills, and within oocytes of female gonads of the blue mussel (*Mytilus galloprovincialis*) [22, 41]. Additionally, under a light microscope, both PS and high-density polyethylene (HDPE) MPs were observed in HE-stained tissues, including digestive glands, gills, gonads, and muscles [42]. However, MP detection with light microscopy has limitations, including the inability to identify polymer types [43] and difficulty detecting transparent MPs [44].

#### Polarized light microscopy for birefringence-based MP detection

Two additional histopathological methods, polarized light and fluorescence microscopy, were used to determine the distribution of MP particles in the tissue samples. The polarized light microscope helped identify and differentiate MPs from other PMs. Analyzing changes in polarization with a positive signal can reveal the unique features of MP particles, which appear as birefringent objects due to their optical properties, chemical makeup, and ability to be detected [25].

In this study, MP-contaminated green mussels showed birefringent features under polarized light microscopy, similar to several studies that have used polarized light microscopy to identify different types of MP contamination in mussel tissues, such as PS in secondary tubules and intestinal lumens of giant mussels (*Choromytilus chorus*) [24], polyvinyl chloride (PVC) and HDPE in digestive glands and gill samples of blue mussels [13, 45], and PE and PS in the digestive guts, digestive glands, gills, and hemolymph samples [46, 47].

This indicated that polarized light microscopy is suitable for MP detection. Moreover, polarized light microscopy is a quick and effective method for detecting MPs, including PE, polypropylene (PP), PS, and polyethylene terephthalate (PET), which are commonly found in green mussels [10], sea surface and beach sand in Rayong Province [20], and wastewater [48].

On the other hand, polarized light microscopy also detected non-MP particles, including dirt particles [49], mussel muscle fibers, opaque and thick MP [50], cellulose fragments and toilet paper pieces [48], and silicates [51].

#### NR fluorescence microscopy for MP confirmation and spectral characterization

Further detection of MP was carried out using NR fluorescence staining. The investigation and characterization of MP particles, especially MP contaminants in tissues and environmental samples, such as sediment, water surfaces, and marine organisms, are quick and common [16, 48, 52, 53].

NR is a hydrophobic dye commonly used in histology that enables the screening for MP when combined with fluorescence microscopy, allowing their visualization. Notably, the solvatochromic properties of NR can also be used to categorize MP based on the surface polarity of different polymer types.

Different wavelengths of fluorescence light, including blue, green, orange, and red, can excite NR-stained MPs. The colors emitted and the signal intensities depend on the types of MPs [54]. The recommended wavelengths for MP detection were 515–565 nm (for green fluorescence from NR-stained MP) and 573 nm (for orange fluorescence from NR-stained MP), because the MP types commonly found in green mussels (PE, PP, and PS) [53] exhibit strong green fluorescence, while other MP types (PET, polyamide (PA), and PVC) do not fluoresce.

Furthermore, PE, PP, and PS exhibit weak orange fluorescence, whereas PET, PA, and PVC exhibit strong orange fluorescence [54]. According to the results, the NR-stained tissue in this study emitted green fluorescent objects with the FITC filter and orange fluorescent objects with the Rhod filter. Additionally, LDPE powder particles in the positive control displayed green fluorescence with the FITC filter and orange fluorescence with the Rhod filter. These results indicated that the contaminated MP could be PE, PP, or PS groups, which are reported to frequently contaminate green mussels in the Gulf of Thailand [10, 53], as these MPs emit strong green fluorescence and weak orange fluorescence, potentially aiding in the identification of MP types under fluorescence microscopy.

On the other hand, various wavelengths of fluorescence light may be affected in field studies, as fluorescence emitted from NR-stained MP can change with different environmental exposure times, colors, chemical components, and MP types [54]. For example, NR-tagged MPs were used in another study, in which PET-type MPs labeled with NR appeared as blue PET particles at excitation wavelengths of 515–560/630 nm in mussel tissues [55].

Furthermore, NR pigment aggregation in adjacent tissues may disrupt histological sections used for MP detection, while NR staining can bind to neutral lipids, lignin, and other organic substances within cells, potentially leading to false-positive results [23, 56].

Notably, the light and polarized microscopes used in the current study showed a higher prevalence of suspected-positive MP samples than the fluorescence microscope with NR stain and the combined trimodal examination. NR-positive signals were lower, which may be due to differences in fluorescence signals from NR-stained MP in the field study [54] and to differences in the solvents used to dissolve NR [57].

Previous research that examined NR-labeled MP under fluorescence microscopy with laser direct infrared spectroscopy showed that NR dissolved in non-polar solvents produced high-intensity fluorescence for hydrophobic MP types, such as PE and PP, whereas NR dissolved in polar solvents generated strong fluorescence for hydrophilic MPs, such as PET and environmentally degraded MPs.

Additionally, the type of solvent used for NR dissolution was highly sensitive to the PS fluorescence signals [57]. In the present study, the NR solvent was dissolved in methanol, a more polar solvent than that used in previous research, potentially leading to a decrease in the fluorescence intensities of PE and PP.

Furthermore, tissue sections contained abundant organic substances, such as cellular structures in the digestive lumens, which might interfere with MP detection [23, 56]. Therefore, the trimodal examination used in the current study proved to be an effective tool for screening and investigating MP contamination in mussel tissues. Nevertheless, each of these methods has its limitations.

A combined histopathological method can improve MP particle detection while reducing false-positive signals. This method was used in another study that employed light microscopy, polarized light microscopy, and fluorescence microscopy to find MP contamination in mussels [24]. Further analysis could combine physical MP characterization methods (trimodal examination) with functional group analysis of MPs, including μ-FTIR or Raman spectroscopy [16] for improved MP detection precision.

In the present study, MP detection methods lagged behind MP type identification, indicating that future analyses should combine trimodal techniques with functional group characterization methods, such as μ-FTIR or Raman spectroscopy, along with histopathological approaches and AI-based image recognition.

This combination could improve MP detection in field studies in Rayong and other coastal areas and enhance understanding of the potential effects of co-localization of various MP types within green mussel and marine organism tissues.

### MP contamination of green mussels

#### Transport pathways of MPs in mussels

In natural environments, MP particles become trapped with food items, filtered through the gills of the green mussel, and then transported into the digestive system. MP particles are internalized by hemocytes via endocytosis in the circulatory system and transported to non-digestive tissues, such as gonadal tissues, adductor muscles, and body cavities. Other studies have identified MP contamination in various tissues, including the digestive tracts, digestive glands, gills, gonadal tissues, adductor muscles, and hemocytes [22, 42, 58].

#### Tissue distribution observed in this study

The current study examined MP contamination in various tissues of the green mussel, with the highest accumulation in the digestive tracts, followed by the gills, digestive glands, body cavities, and male gonads. In contrast, the adductor muscles and female gonads had the lowest MP accumulation.

These findings align with another study that reported MP particles were mainly found in the digestive tracts, followed by the gills, while the lowest accumulation was seen in the gonadal tissues and adductor muscles of blue mussels (*M. galloprovincialis*) after 96 hours of exposure to 50 μg/mL of MP, with the digestive organs and gills serving as the primary sites of environmental MP uptake [42]. The digestive tracts may adapt to particle selection, prolonged buildup, and the excretion of MP particles, which could explain the lower accumulation or absence of MP particles in non-digestive tissues compared to digestive tracts [59].

#### Influence of mussel size on MP contamination

The relationship between green mussel size and MP contamination was analyzed, and detectable MP levels in the samples did not vary significantly across mussel sizes (Table 4). However, the smaller group tended to have higher contamination levels than the larger group, as indicated by a p-value of 0.047 compared with the small- and medium-sized groups (Table 5).

This finding was similar to that of another study that examined small-sized green mussels (2–3.9 cm) and found they had higher percentages of MP contamination than medium-sized (4–5.9 cm) and large-sized (>6 cm) mussels because the higher filtration and pumping rates reduced the MP levels as shell size increased [60].

Nevertheless, another study reported the influence of shell size on MP accumulation in the hard clam (*Meretrix meretrix*; Linnaeus, 1758), where the MP count per individual increased by 1.01 times for every 1 mm increase in shell width [61].

Similarly, in the trigonal clam (*Tivela mactroides*), MP contamination was detected in early life stages, with accumulation increasing with shell length, attributed to a longer exposure period, enhanced filtration activity, and increased retention during growth [62].

#### MP abundance compared with previous regional data

Interestingly, the trimodal examination used in this study revealed that the total number of MP-contaminated mussel tissues cultured in the industrial area was 2(1) particles per section of one mussel sample. Compared with previous studies, MP contamination in mussel tissues using chemical digestion methods was reported as 3.2 ± 1.6 particles per individual in Sriracha Bay (eastern Thailand) and 1.2 ± 0.2 particles per individual in Phetchaburi (western Thailand) [16]. Based on the current MP contamination results, the industrial area and shoreline communities may be contributing to increased MP levels [16]. The sampling site in this study received runoff from the main inland river estuaries, indicating MP transport from mainland sources into the marine environment [20]. Similar findings in Southeast Asia reported MP contamination in mussels using chemical digestion techniques, with 25.05 ± 5.36 particles per individual in Vietnam [63] and 13.5–15.7 particles per individual in Indonesia [64].

These results highlight growing concern about MP contamination in seafood and potential impacts on human health in the region. However, this study was the first to report MP abundance in Thailand using particles per section as the measurement unit. This metric does not directly represent the total MP load per individual or per gram of wet weight in Thailand and therefore does not clearly reflect the overall MP burden in mussel tissues. This limitation arises from histopathology-based MP detection techniques. To improve accuracy and comparability, future research should include comparative MP abundance data obtained through both trimodal examination and digestion-based or spectroscopic methods.

#### MP morphology and environmental sources

Additionally, the main types of MPs in contaminated tissues were fragments and fibers, similar to previous findings in mussels, water, and sediments from Sriracha Bay and Phetchaburi [16]. Moreover, a study in Rayong Province reported that fragment-type MPs predominated in beach sand, whereas fiber-type MPs were mainly found on the water surface [20]. However, investigations of bivalves cultured in eastern Thailand found that fibers were the main type of MP. These variations suggest that MP type in mussel tissues reflects environmental conditions in the surrounding area. Fragments likely come from the breakdown of plastic packaging or containers commonly used in green mussel aquaculture, while fibers probably originate from fishing gear and human activities [16, 20, 21]. Furthermore, the most common MP size range found in this study was 10–100 μm, aligning with previous research on green mussels and other bivalves in Thailand, where MPs generally ranged from 50–300 μm [16] or <1000 μm [10]. These results support reports that mussels selectively ingest smaller MPs and show reduced uptake of 100 μm particles compared with 10 μm particles [65].

#### Need for environmental MP monitoring

This study did not include data on MP contamination in surface water, beach sand, or sediment across different seasons near mussel farms or local fishery markets. Future research should include environmental MP monitoring to identify sources and transport pathways, thereby supporting effective waste management strategies.

### Overview of pathological findings

#### Digestive tract pathology

In the present study, pathological changes were examined in various tissues of green mussels. The tissues with the highest MP contamination were in the digestive tracts, showing no observable histopathological changes, including no alterations in the tubular lumen or tissue destruction within the digestive-tract areas. However, mild lesions with epithelial cell damage and hemocyte infiltration in the connective tissue near the epithelium were observed in only one sample. This supports previous findings of pathological changes in the digestive tracts, including epithelial damage, disruption, elevated intestinal lumen, and necrosis, after exposure to weathered PE [66]. Furthermore, hemocyte infiltration has been observed in the intestinal epithelium of blue mussels (*M. galloprovincialis*) [22] and giant mussels (*C. chorus*) [24].

#### Gill pathology

The second level of tissue contamination was found in the gills, which come into direct contact with MPs in the environment. Pathological lesions were assessed and primarily classified as having no observable histological changes, with no signs of hemolymph sinus thickening or increased size of water tubules. Conversely, one gill sample exhibited mild pathological changes, including cilia destruction and hemocyte infiltration. Similar findings have been reported in clams (*Ruditapes philippinarum*) and scallops (*Chlamys farreri*) exposed to PE and PET particles [67], as well as epithelial loss, gill filament hyperplasia, hemolymph-vessel obstruction, tissue destruction, cilia damage, increased cell numbers, thickened epithelia, and hemocyte infiltration in green mussels after MP exposure [68, 69] and in hard-shelled mussels (*Mytilus coruscus*) [70, 71]. These lesions can affect the gas exchange, food collection, and filtration efficiency of green mussels [66].

#### Digestive gland pathology

Similarly, the digestive glands showed no observable histopathological changes in nearly all MP-contaminated samples. Only two samples showed mild (digestive lumen atrophy with loss of digestive cells) and moderate degrees (lumen destruction and tissue damage with hemocyte infiltration). Previous studies have documented pathological changes, such as epithelial hyperplasia, digestive cell damage, tubular atrophy, and tissue destruction in green mussels, clams (*R. philippinarum*), and scallops (*C. farreri*) [67, 68], as well as hemocyte infiltration in hard-shelled mussels (*M. coruscus*) [70].

#### Lack of pathology in other tissues

No pathological changes were observed in other tissues, including male and female gonads, adductor muscles, and body cavities. This could be because mussels can protect themselves by adapting to particle selection and egesting MP through their digestive tracts, thereby reducing MP translocation to other organs [59].

#### Interpretation of limited pathological findings

Thus, the pathological evaluation results in the current study showed that most samples did not display visible histopathological changes in both macroscopic and microscopic examinations, which might be due to fluctuations in MP concentration in cultivated green mussel areas [72], where waves and currents can decrease MP loads. Similar findings were observed in juvenile oysters (*Crassostrea gigas*), where long-term exposure to low doses of MP beads resulted in MP accumulation in the digestive tracts and glands without eliciting an inflammatory response [73]. Similar results were seen in *Mytilus* spp. and *C. gigas* exposed to PE and PP, with no significant pathological or inflammatory responses detected [74, 75].

#### Mechanistic insights into MP toxicity

The toxicity of environment-contaminated MP could result from various factors, such as MP concentration, exposure duration, MP size, composition, and exposure pathways. Inert particles can cause oxidative stress, immune responses, disrupted gene expression, neurotoxicity, lysosomal dysfunction, and increased cellular and tissue changes [72, 76]. These findings align with previous research [73], which showed no inflammation in mussel tissues. Green mussels can select and expel MP contamination from their bodies. For instance, blue mussels exhibited increased antioxidant enzyme activity, such as superoxide dismutase and catalase, after exposure to PE and PP without showing any physiological responses, significant pathological alterations, or inflammatory responses [74]. Likewise, the Pacific oyster (*C. gigas*) did not exhibit oxidative stress, immune activation, DNA damage, histological changes, or altered clearance rates [75]. These results suggest that green mussels may adapt to MP-contaminated environments. They can selectively filter particles at their gills [74], leading to increased antioxidant enzyme activity and normal clearance rates. MP can also be excreted through the intestine, reducing internal contact as it is transformed into slurry by the digestive system along with mixed food particles [59]. These mechanisms may decrease the duration of MP exposure within the body, facilitate translocation to other tissues, and help minimize tissue damage.

#### Tissue-specific MP effects and functional damage

However, MP co-localization in tissues can cause pathological changes. This study showed evidence of mild to moderate pathological lesions in the digestive tracts, gills, and digestive glands. The epithelial cells in the digestive tract serve as a gut barrier that protects the organism from pathogens and pollutants.

At the subcellular level, the digestive tracts showed lower activity of antioxidant enzymes, including glutathione peroxidase and catalase, while lipid peroxide levels increased, indicating potential oxidative stress that led to intestinal cell damage and inflammation due to reduced energy intake, growth rate, and migration rate of mussels [66]. Moreover, our results indicated that MPs near the epithelium of the digestive tracts might cause direct damage. Lesions in gills, such as cilia destruction and hemocyte infiltration, can impair gas exchange, food collection, and filtration efficiency in green mussels [66]. In addition, hemocyte infiltration reflects a protective immune response in bivalves that shields tissues from foreign objects, including MPs and other pollutants, as part of inflammation and tissue repair [59, 77]. MP exposure can affect immune function by inducing oxidative stress, destabilizing lysosomal membranes, triggering hemocyte apoptosis, and producing more toxic effects when combined with other pathogens than MPs alone [78]. Furthermore, pathological changes in the digestive glands resulted in decreased antioxidant enzyme levels and increased lipid peroxide and hydrogen peroxide levels, potentially triggering oxidative stress and histopathological changes [70]. Damage to the digestive glands can also impair digestive enzyme activity, impacting enzyme function in blue mussels (*M. galloprovincialis*) [79], possibly reducing net energy absorption from food, depleting energy reserves, and ultimately affecting mussel body condition and population size. Additionally, prior studies reported that the digestive glands are the most affected organ, followed by the gills and gonads [68], and that the intestine, followed by the gills, is the most sensitive and targeted organ after exposure to weathered PE in green mussels [66]. This aligns with our current findings, where only the digestive glands exhibited moderate lesions. These results suggest that tissues involved in MP uptake, such as digestive glands, digestive tracts, and gills, could be used to monitor MP exposure in mussels.

### Relevance to human exposure and public health

In Thailand, the estimated MP intake from bivalve consumption was reported to be approximately 0.52 particles/person/day, with an annual exposure ranging from 0.23 to 1178.42 particles/person/year [10, 17], raising concerns over the potential human health implications. Human exposure to MP occurs mainly through ingestion, followed by inhalation and dermal contact [4]. The documented toxicological effects include oxidative stress, immunotoxicity, genotoxicity, neurotoxicity, and cellular damage in human cell models [12]. Additionally, MPs have been shown to absorb plasticizers, metals, and other plastic additives, potentially impacting both bivalves and human health [12, 80]. The histopathological lesions observed in this study provide a useful reference for future research on the potential effects of MP in tissues.

## CONCLUSION

This study successfully applied a standardized trimodal histopathological approach, combining light microscopy, polarized light microscopy, and NR fluorescence staining, to detect, locate, and characterize MPs within *P. viridis* collected from mussel farming areas near industrial zones in Rayong province, Thailand. Using this integrated method, MPs were found in 39 of 61 mussels (63.9%), with the highest prevalence in the digestive tracts (64.1%), followed by gills, digestive glands, body cavities, gonads, and adductor muscles. Most MP particles were irregular fragments (91.67%), mainly in the 10–100 μm size range, and showed birefringence and FITC/Rhod fluorescence similar to LDPE positive controls. Importantly, histopathological examination revealed that most tissues contaminated with MPs did not exhibit observable lesions, although a few cases showed mild to moderate tissue changes, including epithelial damage, hemocyte infiltration, and digestive gland atrophy.

The findings show that histological preservation of MPs is achievable using an isopropanol-based, xylene-free tissue-processing method. The trimodal approach provides a contamination-controlled, cost-effective way to screen for MP in routine diagnostic and ecological monitoring labs, especially in areas without access to advanced spectroscopic tools. Additionally, identifying digestive tracts, digestive glands, and gills as primary MP accumulation sites supports their use as sentinel organs for future biomonitoring efforts.

A major strength is the use of LDPE-spiked positive controls to validate optical signatures across all three imaging modalities, ensuring reliable MP confirmation. The study also represents the first histopathology-based MP investigation in Thai mussels using particles per section as a standardized quantification unit and provides high-resolution localization of MPs within tissue microenvironments.

However, the histopathological approach cannot measure MPs per individual mussel or per gram of tissue, nor can it definitively identify polymer types without additional spectroscopic techniques. Also, the lack of simultaneous environmental sampling (water, sediment, beach sand) restricts the ability to trace contamination sources. Variability in NR fluorescence in field-exposed MPs and solvent-dependent dye interactions may also lead to false negatives.

Future research should combine trimodal imaging with μ-FTIR/Raman spectroscopy and AI-powered image recognition to enhance identification accuracy and polymer classification. Seasonal environmental sampling, hemolymph and mantle assessments, and dose–response exposure studies would help clarify MP transport routes, toxicodynamic effects, and ecosystem risks. Implementing this standardized protocol across multiple coastal provinces would strengthen national MP monitoring and support One Health risk assessments.

Overall, this study shows that MPs are present in aquaculture-relevant *P. viridis* along Rayong’s industrial coast, although pathological effects seem limited at current exposure levels. The validated trimodal histopathological technique offers a strong and practical framework for MP biomonitoring in mussels and other marine organisms. These findings provide valuable evidence for environmental management, food safety assessment, and long-term ecological health monitoring in Thailand’s coastal ecosystems.

## DATA AVAILABILITY

The supplementary data can be made available from the corresponding author upon request.

## AUTHORS’ CONTRIBUTIONS

PK and SN: Conceptualized the study, performed the laboratory examination, and drafted the original version of the manuscript and finalized the manuscript. PK, SN, PC, KS, and PT: Conducted field surveys and assisted in sampling and data collection. PK, SN, TJ, and WS: Analyzed and interpreted the data. All authors have read and approved the final version of the manuscript.
